# Review of "*Non-native and invasive ticks: Threats to human and animal health in the United States *" by Michael J. Burridge

**DOI:** 10.1186/1756-3305-4-78

**Published:** 2011-05-15

**Authors:** Olivier AE Sparagano

**Affiliations:** 1 Northumbria University, School of Life Sciences, Department of Biomedical Sciences, BioReC Centre, Newcastle upon Tyne, NE1 8ST, UK

## Review

This is a unique book dealing with tick species imported to the US through human immigration, migratory birds, exotic animal trade etc. Although the name might suggest it would focus exclusively with ticks found in the US, there are also a few chapters explaining from which continents these invasive ticks were coming and which give valuable information about their ecology, related epidemiology and animal and human health associated problems. The author also presents the different diseases transmitted by these ticks so it is not a taxonomy book *per se*. A special chapter is dedicated to the two most important ticks: *Rhipicephalus (Boophilus) annulatus *and *Rhipicephalus (Boophilus) microplus*. There are also two sections dealing with regulatory issues and preventive measures.

Although it is rather disappointing that the Publisher decided to use only black and white figures, which does not make sense when books are competing against internet animations, colour pictures and other sources. I hope that such a poor choice will be rectified in the second edition. Nevertheless, this book seems ideal for students and scientists interested in ticks and the readership should not be restricted to the US only, The author is a well-known and respected acarologist who has taken an unusual approach to discuss these ticks. It is a book that I am sure many university libraries and acarology centers would find useful as it presents very specific, accurate and up-to-date information.

## Competing interests

The authors declare that they have no competing interests.

